# Comprehensive survey of the truck transportation process: The impact of team driving on health and safety conditions

**DOI:** 10.1016/j.heliyon.2024.e37880

**Published:** 2024-09-12

**Authors:** Hossein Ebrahimi, Shahram Vosoughi, Agha Fatemeh Hosseini, Ali Hatami

**Affiliations:** a Department of Occupational Health Engineering, School of Public health, Iran University of Medical Sciences (IUMS), Tehran, Iran; b Department of Biostatistics, School of Public Health, Iran University of Medical Sciences (IUMS), Tehran, Iran; c Occupational Health Engineering Department, School of Public Health, Hamadan University of Medical Sciences (UMSHA), Hamadan, Iran

**Keywords:** Health and safety conditions, Truck transportation, Human co-driver, Driver assistant, Team driving

## Abstract

**Background:**

Given the limited research conducted on the role of a human co-driver in mitigating occupational health and safety risks, this study aims to investigate the impact of a co-driver on health and safety conditions in the truck transportation process.

**Methods:**

The truck transportation process was divided into three main stages: truck loading, driving, and unloading. Using the job safety analysis (JSA) method, an analysis of the tasks within each stage was conducted, allowing for the identification of essential and safe tasks and conditions. A questionnaire, based on this information, was developed, validated, and used in this study.

**Results:**

The findings of this study demonstrate that a human co-driver positively impacted the drowsiness and alertness levels of truck drivers. Furthermore, improvements were observed in driving and parking performance, alongside a reduction in strenuous tasks and subsequent fatigue. The results conclusively indicate that the presence of a human co-driver significantly enhances health and safety conditions, particularly during the driving stage in comparison to the other stages of the truck transportation process.

## 1 Introduction

In recent years, the annual rate of injuries caused by road incidents has increased. While the rates of death and disability have escalated in developing countries, they have declined in developed nations. However, road injuries continue to rise in developed countries [1]. It is estimated that by 2022, road and traffic crashes will become the third leading cause of global mortality [2]. The risk of death in truck crashes is seven times higher than the average risk in other occupations [3]. According to an analysis conducted by the Federal Motor Carrier Safety Administration, motor vehicle crashes (MVCs) involving trucks have a high severity, particularly for drivers of other vehicles or pedestrians (accounting for 83 percent of all fatalities), due to the substantial weight of trucks [4]. Four factors contribute to the occurrence of MVCs: road conditions, human, vehicle, and environmental factors [5]. It is widely recognized that 80 to 90 percent of incidents are a result of human errors [6]. The truck transportation process consists of three stages: cargo loading, transportation, and unloading. Unsafe behaviors can occur at each of these stages. Truck drivers are consistently exposed to several factors, including heavy workload, long and exhausting driving distances, fatigue, and irregular sleep and rest periods, which have a significant negative impact on their safety and health [[7], [8], [9]].

Most studies focus on human factors in the driving process. Some studies investigating the effects of passengers on drivers reveal that passengers can be sources of distractions, but they can also have a positive impact and even assist with navigation and traffic [[10], [11], [12], [13]].

Issues related to income, job insecurity, and economic conditions can contribute to psychological problems. As a result of these financial difficulties, drivers are unable to hire driver assistants as they lack the necessary financial resources to pay their wages. Therefore, just 13 percent of all truck drivers engage in team driving [14].

In our review of the literature, we found no study investigating the role of human driver assistants (team driving) in reducing incidents and occupational health and safety risks. Most studies are centered around Advanced Driver Assistance Systems (ADAS) [[15], [16], [17], [18], [19], [20], [21], [22], [23], [24], [25], [26]].

Given the aforementioned factors, the primary objective of this study is to investigate the impact of driver assistants (co-drivers or team driving) on the safety and health of the truck transportation process.

In this study, a driver assistant or co-driver refers to an individual who has been hired by a truck driver, possesses a valid driver's license, and is responsible for providing assistance to the driver at all stages of the transportation process.

## 2 Methods

### 2.1 Study population

The present study involved conducting an interview-observational case-control study on a sample of 84 male truck drivers. The drivers were divided into two groups: the case group (n = 37), consisting of drivers with driver assistants, and the control group (n = 37), consisting of drivers without driver assistants. The determination of the sample size was based on data provided by Chen's study [27] and the application of the following statistical formula [28]:

$$n=\frac{2{\left(Z1-\frac{\alpha }{2}+Z1-\beta \right)}^{2}\;p\left(1-p\right)}{{\left(p0-p1\right)}^{2\;}}$$

Taking into account a 95 % confidence interval and an 80 % power of the test, and after inserting the necessary values into the formula, the minimum sample size for each group was determined to be 35 individuals. In practice, this number was increased to 37 participants.

All drivers and driver assistants in both groups were male. The two groups were carefully matched in terms of work experience, health condition, and age of both drivers and driver assistants. The drivers' ages ranged from 35 to 65, with an average age of 39.86. It is worth noting that there were only a few drivers with a driver assistant, making it challenging to find drivers with matched driver assistant to participate in this study. The Bazargan border crossing serves as a crucial frontier post on the overland route between Iran and Europe, with a significant volume of cargo traffic reaching millions of tons. This often results in long queues of cargo trucks waiting to cross the border, spanning several kilometers. Therefore, we specifically invited truck drivers who met our inclusion criteria to take part in this study.

In order to identify drivers with matched driver assistants, we reached out to the border and Customs authority, requesting them to notify us whenever a driver was accompanied by a companion. Subsequently, we conducted interviews with the drivers, and if both the driver and their companion met the study criteria, they were included in the study.

The study inclusion criteria were as follows: drivers needed to have a minimum of 5 years of job experience, the driving distance had to be long (requiring at least one week for transportation), drivers must be self-employed or owner operators (since the majority of truck drivers in Iran are self-employed and not affiliated with any particular company), the driver assistants had to be formally employed by the truck drivers rather than being mere passengers or friends accompanying them, the driver assistants also needed to possess a valid driving license to contribute to the driving tasks, and they must be free from drug addiction and in good overall health (it is important to note that the absence of drug addiction and the health status of all drivers were verified annually through their health cards, which were certified by an occupational medicine center). The type of trailer used in this study was a curtain-side trailer. The study was conducted in the northwestern region of Iran, specifically in West Azerbaijan, at the border of Bazargan city in 2022. The drivers participated in this study voluntarily and provided their full informed consent (a written consent form that was approved by the Ethics Committee of Iran University of Medical Sciences was provided). The research ethics committee approval number for this study is IR.IUMS.REC 1395.9411139005.

### 2.2 Data collection tools

In this study, a researcher-developed questionnaire was utilized to gather data. The design of this questionnaire involved the segmentation of the truck transportation process into three key stages: cargo loading, driving, and cargo unloading. The job safety analysis (JSA) method [29] was used to analyze and identify the tasks associated with each stage. Moreover, the Iran Regulations for Transportation Safety and Driving Crashes, literature reviews, existing driver assessment checklists, and driver interviews were consulted. The SHERPA (Systematic Human Error Reduction and Prediction Approach) method was utilized to analyze human errors that lead to unsafe actions [30]. We aimed to address occupational concerns that both the driver and driver assistant may encounter.

It is important to acknowledge that there are cultural and regulatory disparities in truck driving in Iran compared to other countries. Therefore, the issues, tasks, and conditions addressed in the questionnaire should be carefully reviewed and aligned with local laws and conditions prior to implementation. The Safety and Health Evaluation Questionnaire for the truck transportation process included the following two primary categories:

1. Four observational categories must be completed by the analyst

The analyst is required to complete four observational categories:

The first three categories include:

- Technical inspection of the truck prior to transportation
- Provision of essential tools
- Routine tasks that should be performed during transportation.

In such cases, drivers may exhibit carelessness, forgetfulness, or be pressed for time, thereby compromising the quality of these tasks. If a driver assistant is present, they are likely to be assigned by the driver to carry out these tasks. Furthermore, due to workload, fatigue, lack of sleep, and other factors, drivers may not consistently perform these tasks, making a driver assistant beneficial. The fourth category pertains to driver behavior, with the assumption that team driving may influence driving behavior during the driving stage.

2. Five question style categories should be posed to the drivers: It is presumed that a driver assistant can provide alerts, guidance, and signal other vehicles in crowded traffic, help the driver to perform tasks such as trailer hook-up or parking. To ensure the functionality of trailer pneumatic and electric connections, it is necessary for another person to observe the operation of lights and other systems while the driver tests the functions from inside the truck. Tasks such as opening and closing the curtain side trailer, tire puncture repair, and putting on snow chains, as well as driving for extended periods, can be physically demanding and strenuous, potentially leading to musculoskeletal disorders [31]. Therefore, carrying out these tasks as a team may reduce related adverse effects. Additionally, the presence of a driver assistant can potentially impact alertness, drowsiness, fatigue, and psychological well-being, factors that should be considered when designing the questionnaire. Any comments provided by drivers and driver assistants should be recorded at the end of each category. Furthermore, drivers with a driver assistant were asked about the benefits they experienced, while solo drivers were asked if they perceived any potential benefits of having a driver assistant.

To ensure the quality of the questionnaire, a panel of 10 experts, all holding Ph.D. in occupational health and safety and with published articles in the field of traffic safety, was formed. These experts reviewed the questionnaire for its necessity, simplicity, and comprehensiveness of the questions. Based on their feedback, necessary corrections were made, and the formal and content validity of the questionnaire was established. The questionnaire can be found in Additional File 1.

### 2.3 Data collection procedure

To collect data, the analyst accompanied the truck drivers along the 250 km Tabriz-Bazargan route for approximately 3 h to complete the observational part of the questionnaire. Subsequently, relevant questions were posed to the drivers in order to complete the questionnaire.

Following data collection, a comprehensive literature review was conducted to identify any risks or potential problems associated with deficiencies in sub-tasks, as well as any potential benefits that could arise from the presence of a driver assistant.

### 2.4 Statistical analysis

SPSS version 22 software was utilized to analyze the gathered data using independent *t*-test, chi-square, and fisher tests. The significance level for this study was set at 0.05.

## 3 Results

The study involved the calculation of the content validity ratio (CVR), the mean number of judgments, and the acceptance or rejection outcomes for each item in the questionnaire. The minimum acceptable CVR value, as determined by the Lawshe table [32] was 0.49. The average content validity for all questions was found to be 0.8. The questionnaire's content validity index (CVI) was established as 1, with a minimum CVI index of 0.79. Overall transparency was rated at 0.85, while the overall proportion was determined to be 0.87. Additionally, the overall simplicity of the questionnaire received a score of 0.82. The average value for the total content validity of the questions was calculated to be 0.85. Lastly, the questionnaire was validated using Cronbach's alpha coefficient, which yielded a value of 0.86.

Table 1, Table 2 present an overview of the risks or potential problems arising from deficiencies in sub-tasks, as well as the potential benefits of driver assistant. The information provided in these tables is based on a comprehensive literature review and incorporates the opinions of both drivers and driver assistants.

**Table 1:** The risks, potential problems, and potential benefits associated with driver assistance can be classified into four observational categories.

Task category	Risks or potential problems	Potential benefits of driver assistant
Technical inspection before transportation	Abolishing inspections may increase the occurrence of crashes [33].	According to the driver's notes, certain technical parts of the truck need to be replaced periodically or based on specific driving distances. As a result, regular maintenance is crucial. It is recommended to record the schedule of these services in a notebook to ensure they are not forgotten.
	Visual impairments and crashes caused by brake or turn signal light malfunctions [34].	
	To prevent brake failures, it is recommended to implement double circuit redundancy [35]. Ensuring the proper functioning of the pneumatic system is crucial for the turbo-operated vehicles, as any air leaks in the pipes can compromise the brake performance. Furthermore, the pneumatic system is also connected to the air springs.	
	Environmental factors, such as dirt accumulation on sensor covers, have been found to impact system performance [36].	
Provision of essential tools	The availability of essential tools and equipment plays a critical role in ensuring safe driving practices [37]. Negligence on the part of the driver in performing these tasks must be taken into account.	In order to maintain the equipment, the driver assistant might be assigned by the driver to check, repair, or purchase necessary items (equipment details are provided in the questionnaire).
Routine tasks that should be done during transportation	It is imperative to conduct regular technical inspections before commencing the driving process to verify the proper functioning of all truck components. Any detected defect must be promptly addressed to mitigate potential incidents.	In this case, teamwork would be advantageous. Based on the driver's note, it is important to regularly clean the front grid of the truck during blizzards as snow can accumulate and freeze, preventing air from entering for cooling.
Driver's behavior while driving	Research has established a correlation between aggressive behavior and the frequency of horn honking [38].	According to the driver's note, the driver assistant can have both positive and negative impacts on the driver's behavior.
	Moreover, studies have shown a correlation between anger and the occurrence of near crashes [39].	
	The presence of passengers can have both positive and negative influences on driving behavior [[40], [41], [42]].	
	There is a potential risk associated with In-vehicle information systems (IVIS) use [43] Furthermore, distraction while driving can be caused by various factors [44].

**Table 2:** Risks or potential problems and potential benefits of driver assistant about five questionnaire Categories.

Task category	Risks or potential problems	Potential benefits of driver assistant
Driving tasks	Collision of the truck with the trailer can occur.	Driver assistant can provide guidance and warnings from outside the truck.
		The skill and experience of the driver play a crucial role in fulfilling this task.
	Any misconnection has the potential to lead to an incident.	Another individual is required to confirm that the lights are functioning correctly.
		The technology utilized in modern trucks demonstrates the accurate operation of essential components and is also capable of alerting to potential defects.
	A blind spot can result in collisions with obstacles.	Driver assistant can guide and warn from outside the truck Driver skill and experience play an important role in performing this task
	Fatigue can have a detrimental effect on alertness.	Team driving will be beneficial drivers can travel much more distance in a day because mandatory rests are required less frequently [45]
Other tasks related to truck	Exhausting tasks can lead to both fatigue and musculoskeletal disorders [31].	
	Multitasking can cause distractions and errors [46] especially when dealing with navigation while driving, which can divert the driver's attention.	According to the driver's note, the route suggested by the navigation systems may not be suitable for the dimensions of the truck. Consequently, the truck may encounter difficulty in passing through narrow passages or low-height bridges. To mitigate these challenges, the driver assistant can offer assistance in route planning, selecting appropriate navigation options, and locating specific addresses.
Alertness	Maintaining alertness is crucial for safe driving, as any decrease in alertness can lead to crashes [47]	Based on the driver's note, engaging in conversation with a driver assistant and listening to music can have a negative impact on one's ability to focus on crucial tasks.
		Furthermore, the driver assistant is capable of effectively detecting animals and obstacles on the road and alerting the driver.
Fatigue	Spending long hours on the road can negatively impact sleep quality [48] and drowsiness is a major cause of crashes [49]	Teamwork can affect rest time and reduce exhaustion. According to the driver's note; the driver assistant yawning or sleeping can induce sleepiness in the driver
	Factors contributing to pain are diverse and may include prolonged sitting, poor posture, exposure to whole-body vibration, and non-driving factors such as heavy lifting, poor diet, or other factors [50]	
	crashes caused by driver fatigue [49,51]	
Psychological issues	There is a correlation between psychological health and crashes [53]	Driver assistant have been shown to have the potential to alleviate driver depression [52] and mitigate feelings of loneliness.
	Crashes are associated with dissatisfaction with work conditions [54]	
	Driver security is threatened by security breaches, such as hijackings and other theft crimes [55]	The presence of a driver assistant can enhance the sense of security.
	Economic problems have been found to be linked to increased suicide rates [56]	

Table 3 shows the observational items for the Technical inspection of the truck before transportation, provision of essential tools, routine tasks that should be performed during transportation, and Driver's behavior while driving. As shown in the table, there is a significant difference of two groups only in the routine tasks during transportation.

**Table 3:** Observational items.

Task category	Subtasks	With driver assistant (%)			Without driver assistant (%)			P-value
		A	M	UA	A	M	UA	
Technical inspection of the truck before transportation	Check the lubrication, fuel, oil, and water levels	63.6	27.3	9.10	67.6	32.4	0.00	0.228
	Inspect the lights, tires, wipers, and electrical fuse panel	78.8	21.2	0.00	75.7	18.9	5.40	0.610
	Test the pneumatic system	87.9	12.1	0.00	78.4	18.6	3.00	0.522
	Clean the windows, lights, signs, and mirrors	87.9	12.1	0.00	81.1	13.5	5.40	0.640
Provision of essential tools	Ensure the toolkit set, hand light, and portable warning sign are present	93.9	6.10	0.00	86.5	10.5	3.00	0.677
	Have snow chains, a puncture repair kit, and necessary spare parts	87.9	12.1	0.00	89.2	10.8	0.00	1.000
	Bring hygiene products, detergents, drinking water, and nutrients	81.8	18.2	0.00	89.0	8.00	3.00	0.290
	Hygiene products, detergents, drinking water, and nutrients	87.9	12.1	0.00	91.6	5.4	3.00	0.411
Routine tasks that should be done during transportation	Check the oil and water levels and tire condition	81.8	15.2	3.00	51.4	29.7	18.9	0.020∗
	Examine the pneumatic system, electrical system, lights, and trailer connections	69.7	30.3	0.00	35.1	54.1	10.8	0.005∗
	Clean the lights, license plates, safety signs, windows, and mirrors	87.9	12.1	0.00	54.1	40.5	5.40	0.004∗
Driver's behavior while driving	Use the horn appropriately	69.7	15.2	15.2	56.8	37.8	5.40	0.061
	Avoid verbal aggression	60.6	27.3	12.1	43.2	45.9	10.8	0.300
	Respect the rights of other drivers	78.7	15.2	6.10	67.6	24.3	8.10	0.652
	Driving calmly without dangerous maneuvers	78.8	18.2	3.00	73.0	21.6	5.40	0.907
	Driving between lines	72.7	27.3	0.00	78.4	21.6	0.00	0.781
	Do not use a mobile phone while driving	27.3	48.5	24.2	29.7	37.8	32.4	0.686
	Avoid distractions from music players, navigation systems, etc.	51.5	45.5	3.00	32.4	59.5	8.10	0.276

A (acceptable) M (moderate) UA (unacceptable). The significance of variables was evaluated by the Chi-square and Fisher test and distinguished with an asterisk (∗).

Table 4 shows the measurements of task difficulties when considering whether to do them alone or with the help of a driver assistant and alertness issues. As can be seen in the table, having a driver assistant can reduce the difficulties of these tasks.

**Table 4:** Task difficulties and alertness.

Task category	Subtasks	With driver assistant (%)			Without driver assistant (%)			P value
		E	M	D	E	M	D	
Difficulties of this tasks considering doing alone or with the help of driver assistant	Hooking up the trailer	63.6	33.3	3.00	24.3	56.8	18.9	0.002∗
	Parking at the loading and unloading area	54.5	45.5	0.00	27.0	67.6	5.40	0.031∗
	Checking the trailer's pneumatic and electric connections for correct operation	81.8	18.2	0.00	32.4	54.1	13.5	0.001>∗
	Driving for extended periods of time	57.6	39.4	3.00	29.7	43.2	27.0	0.007∗
	Opening and closing the curtain side trailer	60.6	36.4	3.00	27.0	54.1	18.9	0.007∗
	Repairing tire punctures	27.3	63.6	9.10	8.10	43.2	48.6	0.001∗
	Putting on snow chains	66.7	30.3	3.00	29.7	51.4	18.9	0.005∗
	Repairing mechanical issues	48.5	45.5	6.10	21.6	48.6	29.7	0.012∗
	Planning routes, using navigation, and finding addresses	33.3	48.5	18.2	29.7	35.1	35.1	0.278
		A	O	ST	A	O	ST	
Questions about alertness considering being alone or with presence of driver assistant	Ensuring accurate navigation and avoiding route mistakes	78.8	21.2	0.00	59.5	29.7	10.8	0.100
	Paying full attention to traffic signs	78.8	18.2	3.00	59.5	35.1	5.40	0.243
	Paying full and timely attention to vehicle dashboard warning signs and possible unusual sounds indicating technical defects	84.8	15.2	0.00	73.0	18.9	8.10	0.324
	Always feel alert	48.5	42.4	9.10	24.3	35.1	40.5	0.006∗

E (easy), M (moderate), D (difficult). A (always), O (often), ST (sometimes). The significance of variables was evaluated by Chi-square and Fisher test and distinguished with an asterisk (∗).

Table 5 presents the values of fatigue and psychological issues. As shown in the table, nearly all dimensions exhibit a significant difference between the two groups, except for headache, whole body pain, back pain, and stress and anxiety.

**Table 5:** Fatigue and psychological issues.

Task category	Subtasks and occupational and health condition	**With driver assistant (%)**			Without driver assistant (%)			P value
		R	ST	U	R	ST	U	
Fatigue	Feel sleepiness while driving	48.5	45.5	6.10	21.6	48.6	29.7	0.012∗
	Feel a headache	42.4	45.5	12.1	43.2	43.2	10.8	1.000
	Feel whole body pain	57.6	39.4	3.00	48.6	40.5	10.8	0.462
	Feel low back pain	33.3	48.5	18.2	29.7	35.1	35.1	0.278
	Feel tired	45.5	45.5	9.00	18.9	45.9	35.2	0.010∗
Psychological issues	Feel depressed	51.5	39.4	9.10	10.8	56.8	32.4	0.001>∗
	Feel stress or anxiety	54.5	33.3	12.1	45.9	35.1	18.9	0.650
	Feel dissatisfaction with work conditions	33.3	51.5	15.2	8.1	67.6	24.3	0.037∗
	Feel insecurity	84.8	12.1	3.00	45.9	43.2	10.8	0.002∗
	Feel loneliness	51.5	39.4	9.1	16.2	64.9	18.9	0.007∗
	Encounter economic and financial problems	48.5	36.4	15.2	21.6	45.9	32.4	0.043∗

R (rarely), ST (sometimes), U (usually). The significance of variables evaluated by Chi-square and Fisher test and distinguished with an asterisk (∗).

## 4 Discussion

This study aimed to investigate the impact of driver assistant on the health and safety of the truck transportation process. The study findings revealed that, in terms of the technical and mechanical inspection of the truck prior to driving, as well as the provision of necessary tools and materials for travel, drivers with a driver assistant showed slight improvement compared to those without, but this difference was not statistically significant. However, drivers with driver assistant demonstrated significant improvements in areas such as parking, driving, alertness, drowsiness, and distractions. Furthermore, the driver assistant was found to enhance the process of opening and closing the curtain side trailer, attaching wheel chains on the tires, and repairing tire punctures.

According to various studies, several factors including analysis, investigation, forecasting, prevention, and identification and replacement of defective parts play a crucial role in preventing failures [57]. Experienced drivers understand the importance of having essential tools and materials for long-distance driving, and they typically plan and prepare accordingly [58]. Regardless of whether they have a driver assistant or not, experienced drivers recognize the need to address these defects, perform necessary technical services, and ensure the availability of required supplies and equipment. However, routine and repetitive tasks such as technical inspections and cleaning of lights and windows, which are often carried out by driver assistants, can be frustrating for the drivers, considering the already tiring nature of driving.

Research has demonstrated that vehicle technical issues and maintenance can contribute to MVCs [59]. In fact, technical failures of vehicles account for 15 % of road crashes in Iran [60]. Therefore, neglecting routine technical examinations during travel may result in MVCs. New technologies in trucks aid drivers in identifying potential defects through warnings displayed on the dashboard. Nevertheless, potential false alarms should be taken into consideration, and drivers should regularly check the sensors for possible faults. Utilizing a checklist for routine checks and maintaining a notebook to record the dates and mileage of periodic technical and mechanical services can prove useful in preventing oversight.

The study results indicated no significant differences in driver behavior. However, it is worth noting that the presence of the researcher next to the driver during data collection may have influenced these observational items. Therefore, future studies should consider such factors in order to obtain more accurate and unbiased results.

Human driver assistants, such as Advanced Driver Assistance Systems (ADAS), can contribute to drivers in parking and hook up the truck to the trailer. These assistants provide warnings and signals to other drivers, contributing to safer and more efficient maneuvers. Automatic parking systems and alert systems are particularly effective in detecting obstacles and issuing timely alerts to prevent collisions. Additionally, they employ emergency brake systems to minimize the risk of collisions, facilitate parking, and enhance visibility around the vehicle by utilizing cameras that cover blind spots of trucks [61,62].

The findings of this study indicate that the presence of a driver assistant can significantly improve parking efficiency and reduce the occurrence of collisions with obstacles. It is worth noting, however, that drivers still rely on their experience and skills when it comes to parking and hookup the trailer. Moreover, they require assistance in checking the pneumatic and electric connections of the trailer to ensure their correct operation.

Long-distance driving induces fatigue and drowsiness, thereby diminishing driver alertness. According to research, "Impairment (Fatigue, Alcohol, Illness, etc.)" ranked as the second most common cause of MVCs [4]. Long-haul journeys often result in both physical and mental fatigue. Among truck drivers, common causes of MVCs include fatigue, drowsiness, and lack of attentiveness [[62], [63], [64], [65], [66]]. Driver assistant can alleviate physical and mental fatigue by sharing tasks, engaging in conversation with drivers, and reducing drowsiness. However, we couldn't find information on the effects of driver assistant on crash risk.

Sleep diagnostic and driver warning devices can detect driver drowsiness and alert the driver through auditory alarms. This system is known as the Drowsiness Awareness System (DAS) [67] Lane departure warning systems [68] and Automatic Emergency Braking (AEB) [69] are effective in reducing the likelihood of potential crashes. Driver assistant can also fulfill a similar role by promptly notifying drivers of obstacles or lane deviations when the driver is distracted or inattentive. However, the reliability of human driver assistant varies in comparison to Advanced Driver Assistance Systems (ADAS).

Driver assistant can assume driving responsibilities during drivers' designated bedtime or through a team driving schedule, allowing drivers ample time for sleep and rest. Consequently, driver drowsiness, a primary contributor to MVCs, is mitigated. Sleep deprivation compromises consciousness, concentration, and increases errors [70,71]. By reducing driver drowsiness and mental fatigue, a driver assistant can heighten alertness and decrease MVCs. According to the driver's note; when the driver assistant falls asleep while the driver is in control, the driver unconsciously experiences sleepiness. Since yawning is contagious [72], the driver may also feel drowsy when observing the driver assistant yawning or sleeping.

Driver distraction is a notable factor in MVCs. Distractions faced by drivers may arise from various sources, including mental fatigue, mobile phone usage, eating and drinking while driving, using music players, adjusting seats, managing the air conditioner, and organizing equipment while driving. The prevalence of mobile phone usage while driving is particularly high in Iran [73]. Consequently, the presence of an individual who can perform ancillary tasks during driving can reduce the occurrence of MVCs. However, engaging in conversation with drivers may serve as a distraction and increase the likelihood of crashes.

Participating in a task or working as a team can enhance performance [74,75]. Therefore, when carrying out activities such as opening and closing curtain side trailer and repairing tires, the driver assistant's assistance and involvement in these activities can improve driver performance. Adopting an inappropriate posture during the opening and closing of curtain side trailers can also contribute to the development of musculoskeletal disorders [76,77]. Furthermore, this task involves working at heights, which increases the risk of falling. Driver assistants can mitigate such risks by providing support and working as a team [78].

In some loading and unloading stations or warehouses, there is no suitable and standard ground-level entry point for lift trucks to access the trailers easily through the main door. As a result, loading and unloading must be done from the side of the trailer. To facilitate this, the driver needs to fully open the curtain side and secure it on the roof of the trailer using a rope or other ways, so that the lift truck can perform the loading or unloading. In such situations, there is a possibility of a collision between the lift truck and the driver, or even the truck and cargo may fall. Therefore, it is essential to use cargo restraint straps or fastening straps after loading.

Tire repair and chain locking are activities associated with occupational health and safety risks. These risks include physical strain on muscles, finger frostbite due to putting on snow chains, improper posture, lifting heavy loads, prolonged periods of static physical activity, fatigue, and the possibility of collisions with passing vehicles. The assistance of a driver assistant significantly improves performance in all of these tasks. The presence of a driver assistant allows for task sharing and a reduction in applied stress. Additionally, by warning other drivers of passing vehicles, they can play a crucial role in reducing collisions with the truck during tire repair or snow chain application. Working as a team with the driver, the co-driver is more effective and safer than the driver working alone when performing auxiliary tasks such as unloading or truck maintenance.

Depression is a common issue among truck drivers. The age of truck drivers is a significant factor in the risk of depression. Changes in socioeconomic status impact the severity and prevalence of depression. Compared to self-employed truck drivers, wage-earning drivers may be at greater risk of depression due to lower wages and longer work hours [79]. In our previous study [52], we discovered that the presence of a driver assistant can lead to decreased job stress, reduced feelings of loneliness among drivers, increased work performance, and job satisfaction, ultimately resulting in lower job-related depression.

Just like in many countries around the world, trucking in Iran is predominantly carried out by single drivers. Similarly, in Europe, the majority of trucking is also done by single drivers, while team driving being implemented only in specific situations, such as security concerns [45]. The inclusion of driver assistants can prove to be advantageous in the trucking operations, particularly for older drivers [80]. In recent years, there has been a significant surge in truck driver shortages across the Americas, Asia, and Europe, with an increase of up to 40 % observed in 2022. This shortage greatly hampers the practical implementation of team driving [81].

## 5 Conclusion

In this study, we conducted an assessment of the impact of driver assistant on the health and safety aspects of the truck transportation process. Based on the findings, it can be inferred that the inclusion of a driver assistant throughout the transportation process effectively enhances the occupational health and safety conditions. Given the absence of regulations for team driving in most countries, we recommend that authorities and transportation companies consider implementing team driving or driver assistance. Team driving offers the benefit of increased driving time per day, in contrast to the mandatory driving breaks imposed on individual drivers. Furthermore, drivers have the option to personally engage an assistant for long-distance trips. For future research, we propose that scholars undertake a comparative evaluation of human driver assistants and Advanced Driver Assistance Systems (ADAS) to identify the respective advantages and disadvantages associated with each.

## 6 Impact assessment of driver assistants on truck transportation process

This article analyzes the impact of driver assistants on the health and safety of truck drivers. Here's a breakdown of the key points and an assessment framework you can use for further evaluation:

Benefits of Driver Assistants:

• **Improved Alertness and Reduced Fatigue:** Driver assistants can help by sharing driving duties during rest periods (team driving), alerting drivers to potential hazards, reducing drowsiness, and engaging in conversation, mitigating loneliness and mental fatigue.
• **Enhanced Safety:** Assisting with parking and maneuvering, reducing collision risks, warning drivers of lane departures or obstacles sharing tasks like opening trailers and repairing tires, minimizing distractions.
• **Improved Task Performance:** Sharing the burden of repetitive tasks like inspections and cleaning, and assisting with tire repairs and snow chain application, reducing physical strain.
• **Reduced Psychological Issues:** Alleviating feelings of loneliness and isolation, and potentially reducing job-related depression.

Uncertainties and Potential Drawbacks:

• **Limited Impact on Pre-Trip Inspections:** Experienced drivers likely prioritize these tasks regardless of an assistant.
• **Driver Behavior:** The study acknowledges the potential influence of the researcher on observed behavior.
• **Distraction:** Conversation with assistants might distract drivers in some situations.
• **Reliance on Assistants:** Drivers may become overly reliant on assistants, potentially impacting their own skills.
• **Cost and Availability:** Driver assistants may not be readily available or affordable for all drivers.
• **Human vs. Technology:** The study suggests further research comparing human assistants with Advanced Driver Assistance Systems (ADAS).

Assessment Framework:

To make an informed decision about the use of driver assistants, consider these factors:

• **Safety:** How will driver assistants contribute to accident prevention and driver well-being?
• **Cost-Effectiveness:** Weigh the cost of employing assistants against potential savings from accident reduction and improved productivity.
• **Regulations:** Are there legal considerations for team driving or employing assistants in your region?
• **Driver Preferences:** Do drivers find assistants helpful?

Further Research:

The study proposes further investigation into:

• **Comparative Analysis:** Comparing human assistants with ADAS to identify their strengths and weaknesses.
• **Global Considerations:** Exploring how cultural norms, regulations, and driving conditions impact the effectiveness of driver assistants and transportation process.

By carefully considering these points and conducting further research tailored to your specific context, you can make an informed decision about the potential benefits and drawbacks of employing driver assistants in the truck transportation industry.

## 7 Limitations of the study

One of the limitations of this study is the difficulty in finding drivers with driver assistants who meet the inclusion criteria, as the majority of drivers work alone. Additionally, other factors that should be considered are the variations in driving behavior, welfare amenities, cultural norms, regulations, and driving conditions of Iranian drivers compared to those of drivers in other countries. These differences can be further examined in future studies. It is worth noting that in Iran, there is a limited number of cargo transportation companies, and the majority of drivers are self-employed. Consequently, there is little institutional motivation to carry out such studies.

## Data availability statement

All data generated or analyzed during this study are included in this published article.

## CRediT authorship contribution statement

**Hossein Ebrahimi:** Writing – review & editing, Writing – original draft, Supervision. **Shahram Vosoughi:** Writing – review & editing, Writing – original draft. **Agha Fatemeh Hosseini:** Formal analysis, Data curation. **Ali Hatami:** Writing – review & editing, Writing – original draft, Supervision.

## Declaration of competing interest

The authors declare that they have no known competing financial interests or personal relationships that could have appeared to influence the work reported in this paper.
